# Observation of Zeeman effect in topological surface state with distinct material dependence

**DOI:** 10.1038/ncomms10829

**Published:** 2016-02-24

**Authors:** Ying-Shuang Fu, T. Hanaguri, K. Igarashi, M. Kawamura, M. S. Bahramy, T. Sasagawa

**Affiliations:** 1 School of Physics and Wuhan National High Magnetic Field Center, Huazhong University of Science and Technology, Wuhan 430074, China; 2 RIKEN Center for Emergent Matter Science, Wako, Saitama 351-0198, Japan; 3 Materials and Structures Laboratory, Tokyo Institute of Technology, Yokohama, Kanagawa 226-8503, Japan; 4 Department of Applied Physics, University of Tokyo, Bunkyo-ku, Tokyo 113-8656, Japan

## Abstract

Manipulating the spins of the topological surface states represents an essential step towards exploring the exotic quantum states emerging from the time reversal symmetry breaking via magnetic doping or external magnetic fields. The latter case relies on the Zeeman effect and thereby we need to estimate the *g*-factor of the topological surface state precisely. Here, we report the direct observations of the Zeeman effect at the surfaces of Bi_2_Se_3_ and Sb_2_Te_2_Se by spectroscopic-imaging scanning tunnelling microscopy. The Zeeman shift of the zero mode Landau level is identified unambiguously by appropriately excluding the extrinsic effects arising from the nonlinearity in the band dispersion of the topological surface state and the spatially varying potential. Surprisingly, the *g*-factors of the topological surface states in Bi_2_Se_3_ and Sb_2_Te_2_Se are very different (+18 and −6, respectively). Such remarkable material dependence opens up a new route to control the spins of the topological surface states.

## Abstract (web summary)

The knowledge of how electrons behave under magnetic field provides inherent information for exotic quantum states. Here, Fu *et al*. find different *g*-factors of topological surface states in Bi_2_Se_3_ and Sb_2_Te_2_Se, which suggests possible control of such states in spin-related applications.

## Graphical abstract

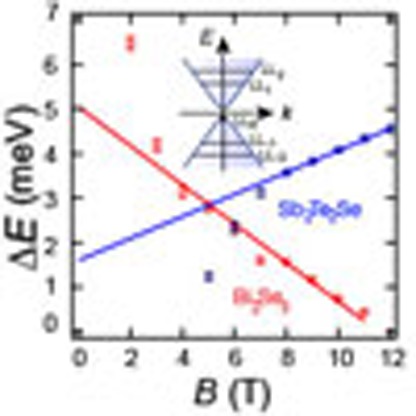

The helical Dirac fermions on the surfaces of topological insulators (TIs) host novel relativistic quantum phenomena in solids^1^,^2^. When the time reversal symmetry (TRS) of the topological surface state (TSS) is broken, a gap opens at the Dirac point. This brings about novel topological excitations, such as the magneto-electric effect^3^,^4^, the quantum anomalous Hall effect^3^,^5^ and the magnetic monopole effect^6^. Magnetic doping has proven to be an effective way to break the TRS via magnetic exchange interactions^7^,^8^,^9^, thereby enabling the experimental observation of the quantum anomalous Hall state^10^. However, magnetic dopants may introduce charge inhomogeneity^11^ and weaken the spin–orbit coupling of TI compounds^12^.

The Zeeman effect, the coupling of spins with a magnetic field, offers an alternative way to break the TRS of the TSS without causing any of the above issues. The opened gap Δ is fully tunable by a perpendicular magnetic field *B*, that is, Δ=*g*_s_*μ*_B_*B*, where *g*_s_ is the electron *g*-factor of the TSS and *μ*_B_ is the Bohr magneton^13^. Consequently, to manipulate the TSS via the Zeeman effect, it is crucial to know its *g*_s_.

Under *B*, Landau levels (LLs) are formed as a result of the cyclotron motion of the electrons. Inclusion of the Zeeman effect can lead to an energetic shift of the LLs as the corresponding TSS lack spin degeneracy (Fig. 1a). This is in stark contrast to the Zeeman splitting of the LLs observed in graphene^14^ and conventional two-dimensional (2D) electron systems^15^. Such a Zeeman effect is most pronounced for the zeroth LL (LL_0_), and decreases dramatically with increasing Landau index *n* (Supplementary Note 1).

Understanding the Zeeman shift behaviour and determining the *g*-factor of the TSS have entailed intense research investigations primarily by the means of quantum oscillation measurements^13^,^16^,^17^. However, the reported data are still largely controversial. The inconsistency is due to the fact that the host materials are inevitably doped by defects. This prevents the lower LLs, which exhibit prominent Zeeman shifts, from reaching the Fermi level and contributing to quantum oscillations in the accessible *B* (ref. 16). Furthermore, the sign of *g*_s_ cannot be determined from the Zeeman shift of the nonzero LLs^17^. A recent tunnelling spectroscopy study appears to be successfully probing the LL_0_ of the TSS formed at an interface with a conventional semiconductor^18^. However, the method used depends on specific details of band-bending in such heterostructures, and therefore cannot be readily applied to other TI compounds. Spectroscopic imaging scanning tunnelling microscopy (SI-STM) can access electronic states in a wide energy range with high spatial and energy resolutions. Thus, it can be used to study any LL, regardless of the doping level of the TI compounds.

Using this technique, we have developed a methodology to unambiguously observe the Zeeman shift of LL_0_. This allows us to determine the *g*-factors of the TSSs in Bi_2_Se_3_ and Sb_2_Te_2_Se precisely, which turn out to be very different.

## Results

### Modelling the Zeeman effect of the TSS

**Figure 1:**
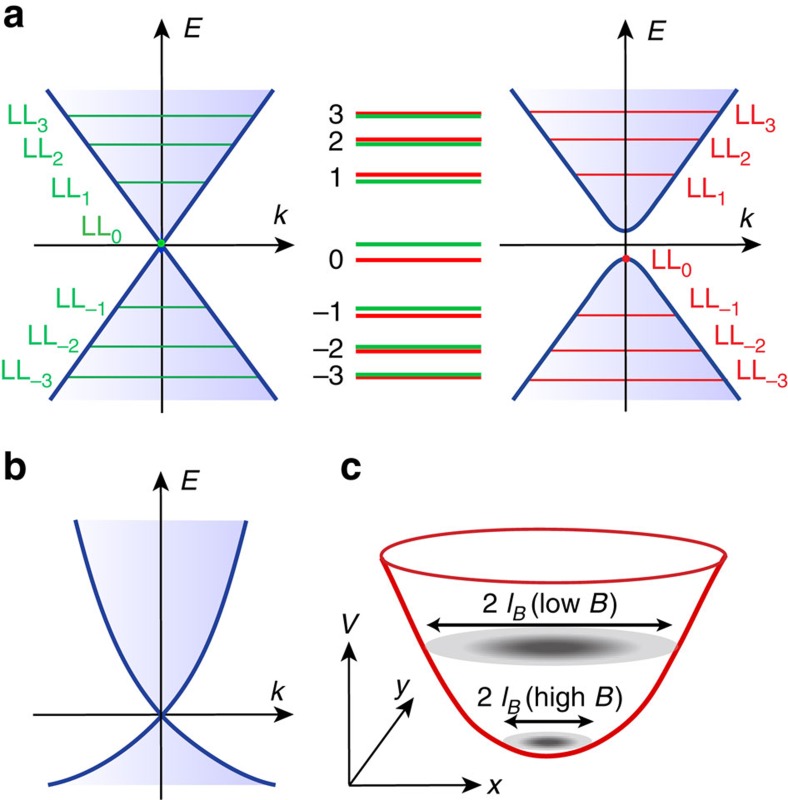
Zeeman effect of TSS and extrinsic influences on its observation. (**a**) Schematic illustrating the Zeeman effect on the LLs of the TSS. When the Zeeman effect is absent, LLs (green lines) are formed in a perpendicular *B*. When the Zeeman effect is present, the TSS becomes massive and its LLs (red lines) exhibit an additional energy shift away from the Dirac point. The magnitude of the Zeeman shift decreases rapidly with increasing Landau index *n*. (**b**) Schematic showing the band structure of the actual TSS with a finite curvature superimposed on its linear dispersion. (**c**) Schematic of a 2D potential minimum and the spatial extension of the LL_0_ wave function at different *B*. The dark (light) grey colour depicts high (low) intensity of the LL_0_ wave function.

In principle, the Zeeman shift of the LL_0_ energy (*E*_0_) of the TSS is linear with *B*, and its slope determines *g*_s_. In practice, however, more factors are involved, hindering its direct observation. On one hand, a finite curvature is superimposed on the linear dispersion of the TSS in actual compounds (Fig. 1b). This induces an extra *B*-linear change in *E*_0_ that is irrelevant to the Zeeman effect. On the other hand, there exists spatial potential variations in the TSS originating from the inhomogenously distributed charged defects^19^. This introduces an extrinsic *B* dependence of LL energies as the spatial extension of the LL wave functions shrinks with increasing *B* (Fig. 1c).

To quantify the effects of nonlinear dispersions and the potential variations, we consider the following model Hamiltonian^17^.


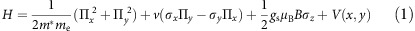


Here, **σ** are the Pauli matrices and **II** are the canonical momenta. The first term denotes the nonlinearity of band dispersions, with m* being the effective mass relative to that of the free electron (*m*_e_). The second term represents the helical Dirac component of the TSS, with *v* being the electron velocity. The third term is the Zeeman term and the last term represents the potential variation. The energy of the *n-*th LL, *E*_*n*_, has been given in ref. 20, albeit without contribution from the last term of equation (1).

To include the effect of this term, we consider a 2D parabolic potential model 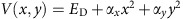 to approximate the shape and location of the potential extremes, where *E*_D_ is the Dirac-point energy. At the potential extreme, the *B*-dependence of *E*_0_ can be calculated using a first-order approximation^21^ (Supplementary Note 1). Including the effects of nonlinear dispersions and potential variations, the *E*_0_(*B*) is accordingly given as


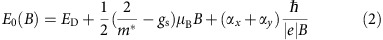


Note that m* renormalizes *g*_s_, and the potential variation introduces an additional 1/*B* dependence term. To determine the intrinsic *g*_s_, we have developed an analysis scheme for our SI-STM data, which corrects these extrinsic factors, m* and *α*_*x*_+*α*_*y*_, and applied it to two different TI materials, Bi_2_Se_3_ and Sb_2_Te_2_Se.

### SI-STM study of the Zeeman effect of the TSS

**Figure 2:**
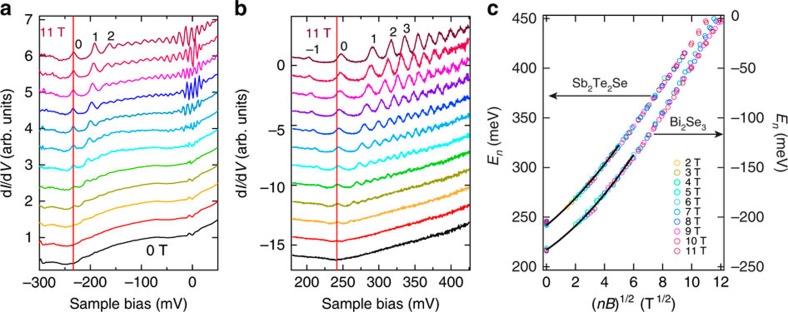
LL spectroscopy of Bi_2_Se_3_ and Sb_2_Te_2_Se. Tunnelling spectroscopy showing the LLs of the TSS measured at a fixed location of (**a**) Bi_2_Se_3_ and (**b**) Sb_2_Te_2_Se surface at 1.5 K. The spectra were acquired at different *B* from 0 T to 11 T with an interval of 1 T and are offset vertically for clarity. The red lines mark the energies of the Dirac points. The data shown in **a** are the same data used in ref. 22. Measurement conditions of **b**: *V*_s_=−100 mV, *I*_t_=50 pA and *V*_mod_=1.4 mV_rms_. (**c**) Scaling analysis of *E*_*n*_(*B*) based on the data of Sb_2_Te_2_Se shown in **b** and a comparison with that of Bi_2_Se_3_. The *E*_*n*_ values were obtained by fitting the LL spectra with multiple Lorentz functions. The black curves depict the fitting to the low-energy parts of the scaling relations.

As the first step, we evaluate m* using momentum-resolved LL spectroscopy^22^. Figure 2a,b display the LL spectra of Bi_2_Se_3_ and Sb_2_Te_2_Se (Supplementary Note 2, Supplementary Figs 1and 2), respectively, as measured at a fixed location at various *B*. In contrast to the electron-doped Bi_2_Se_3_, Sb_2_Te_2_Se is hole-doped and thus its Dirac point is in the empty state. For each material, the corresponding *E*_*n*_ exhibits a quasi-linear scaling relation with a scaling variable (*nB*)^1/2^ (Fig. 2c), which represents the energy–momentum dispersion of the TSS^22^. The potential effect on *E*_*n*_ is more significant for the LLs with small *n* exposed to high *B* than those with large *n* exposed to low *B* (ref. 21). In this regard, the observed scaling with (*nB*)^1/2^ demonstrates that such potential effect has negligible influence on it at the measured location. For both compounds, a finite curvature is evidently seen in the dispersions indicating that m* is finite, as also demonstrated by angle-resolved photoemission spectroscopy measurements and band calculations^7^,^23^. Remarkably, both compounds show a very similar band curvature, despite their different constituent elements. We thus expect m* to be nearly the same for both the compounds. To evaluate this, m* is extracted from the scaling function, while ignoring the potential variations. For each compound, we have performed three measurements at different locations/samples. On the basis of these measurements, m* is found to be 0.12±0.03 for Bi_2_Se_3_ and 0.13±0.02 for Sb_2_Te_2_Se with negligible inhomogeneity.

**Figure 3:**
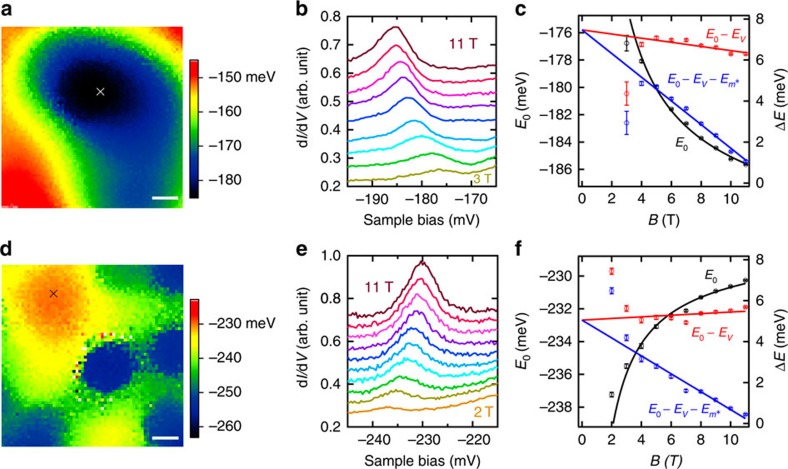
Surface *g*-factor measurement on Bi_2_Se_3_. (**a**) Potential landscape of Bi_2_Se_3_ obtained by mapping *E*_0_ at 11 T showing a potential minimum. The centre of the potential minimum is determined by the 2D parabolic potential fitting and is marked as a cross. The scale bar corresponds to 10 nm. Measurement conditions: *V*_s_=50 mV, *I*_t_=50 pA, *V*_mod_=2.8 mV_rms_ and *T*=1.5 K. (**b**) Tunnelling spectra taken at the potential minimum centre at fields from 3 to 11 T with 1 T intervals. The spectra have been shifted for clarity. Measurement conditions: *V*_s_=−220 mV, *I*_t_=100 pA, *V*_mod_=1.4 mV_rms_ and *T*=1.5 K. (**c**) *E*_0_ at different *B* obtained by fitting the data of **b** with a Lorentz line shape and plotted with black symbols (left axis). The error bars are the standard deviation of the fitting analysis. The effects of the potential and the non-ideal dispersions on the LL_0_ energies are represented by 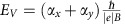 and 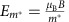, respectively. Their influences can be excluded by subtracting their contributions. The red symbols denote the LL_0_ energies after subtracting the effect of the potential (*E*_0_−*E*_*V*_). The blue symbols correspond to the LL_0_ energies after subtracting the effects from both the potential and the non-ideal dispersions (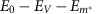) (right axis, in the relative energy scale). The black curve denotes the fitting to *E*_0_ with *B* according to equation (2). The red and blue lines show the linear fitting of *E*_0_−*E*_*V*_ and 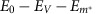
with *B*, respectively. (**d**–**f**) Similar data and analysis as **a**–**c** for a potential maximum of Bi_2_Se_3_. Measurement conditions of **d** and **e**: *V*_s_=−200 mV, *I*_t_=165 pA, *V*_mod_=1.8 mV_rms_ and *T*=1.5 K.

Next, we assess the impact of potential variations on *E*_0_(*B*). The spatial variation of *E*_0_ represents the potential landscape, although it is smeared out by the magnetic length *l*_*B*_ (refs 19, 21). We start with Bi_2_Se_3_ and map out the potential landscape by performing a spectroscopic imaging of *E*_0_ at a high *B* of 11 T, where *l*_*B*_∼7.7 nm (Fig. 3a,d). We focus on areas around the potential extremes (Fig. 3a,d) and fit their shapes with the 2D parabolic potential model introduced above (Supplementary Fig. 3). After positioning the tip at the fitted potential centre (Fig. 3a,d, cross point), the *B* dependence of the LL_0_ peak is measured (Fig. 3b,e). The measured *E*_0_(*B*) is plotted in Fig. 3c,f (black symbols). Intriguingly, *E*_0_(*B*) exhibits ∼1/*B* behaviours at both the potential minimum (*α*_*x*_>0,*α*_*y*_>0) and maximum (*α*_*x*_<0,*α*_*y*_<0), but shifts towards opposite directions. This is exactly expected from equation (2) and directly highlights the influence of potential variations on *E*_0_(*B*).

To eliminate the potential effect, we estimate the last term of equation (2) using the fitted values of *α*_*x*_ and *α*_*y*_. After subtracting the estimated potential effect, we obtain a nearly *B* independent *E*_0_ in the high *B* region at both the potential minimum and maximum (red symbols in Fig. 3c,f). This validates our methodology and indicates that the effect of m* and the Zeeman effect contribute oppositely with an accidental cancellation. By further subtracting the contribution from m*, we obtain the genuine Zeeman shift (Fig. 3c,f, blue symbols). Subsequently, *g*_s_ in Bi_2_Se_3_ is determined to be +18±4. (The error in *g*_s_ is the propagation of uncertainty evaluated from the errors of m* and 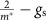, which are extracted above and measured at different potential extremes, respectively.) This differs significantly from its corresponding bulk value +32 as indicated by magneto-transport and nuclear magnetic resonance measurements^24^,^25^.

**Figure 4:**
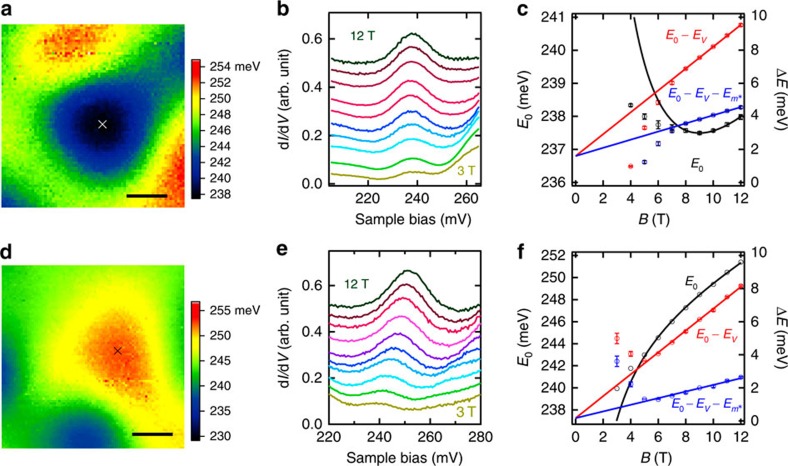
Surface *g*-factor measurement on Sb_2_Te_2_Se. (**a**) Potential landscape of Sb_2_Te_2_Se obtained by mapping *E*_0_ at 12 T showing a potential minimum. The centre of the potential minimum is determined by the 2D parabolic potential fitting and marked by a cross. The scale bar corresponds to 10 nm. (**b**) Tunnelling spectra taken at the potential minimum centre at fields from 3 to 12 T with 1 T intervals. The spectra have been shifted for clarity. (**c**) *E*_0_ values at different *B* obtained by fitting the data of **b** with a Lorentz line shape and plotted with black symbols (left axis). The error bars are the standard deviation of the fitting analysis. The effects of the potential and the non-ideal dispersions on the LL_0_ energies are represented by 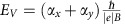 and 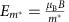, respectively. Their influences can be excluded by subtracting their contributions. The red symbols denote the LL_0_ energies after subtracting the effect of the potential (*E*_0_−*E*_*V*_). The blue symbols correspond to the LL_0_ energies after subtracting the effects from both the potential and the non-ideal dispersions (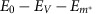) (right axis, in the relative energy scale). The black curve denotes the fitting to *E*_0_ with *B* according to equation (2). The red and blue lines represent the linear fitting of *E*_0_−*E*_*V*_ and 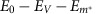 with *B*, respectively. (**d**–**f**) Similar data and analysis as **a**–**c** for a potential maximum of Sb_2_Te_2_Se. Measurement conditions of **a** and **d**: *V*_s_=215 mV, *I*_t_=50 pA, *V*_mod_=2.8 mV_rms_, *T*=4.4 K and *B*=12 T. Measurement conditions of **b** and **e**: *V*_s_=210 mV, *I*_t_=50 pA, *V*_mod_=1.8 mV_rms_ and *T*=4.4 K.

The same methodology is applied to Sb_2_Te_2_Se (Fig. 4; Supplementary Figs 4 and 5). Distinct from Bi_2_Se_3_, the LL_0_ state measured at the potential minimum (Fig. 4b) exhibits a nonmonotonic *B* dependence (Fig. 4c) and increases with *B* at high fields. Given that the effective masses of both compounds are almost the same, *g*_s_ in Sb_2_Te_2_Se must differ significantly from that of Bi_2_Se_3_. Indeed, we evaluate *g*_s_ in Sb_2_Te_2_Se to be −6±2. While both systems share very similar band dispersions in their TSSs, their *g*-factors turn out to be strikingly different in both size and sign.

## Discussion

We elucidate the above results under the framework of **k**·**p** theory. For narrow gap semiconductors, the conduction bands are coupled to the spin–orbit-split valence bands through a second-order perturbative term. Such a coupling substantially enhances the orbital sector of the *g*-factor. As a result, the total *g*-factor of such electrons/holes can significantly deviate from that of the free electrons in both magnitude and sign^26^. In the case of TIs, the strong atomic spin–orbit coupling creates a symmetry-inverted band gap. Thus, the atomic orbital characters of the wave function undergo a strong variation at and in the vicinity of the inverted band edges^27^. Such a variation manifests itself in the orbital character of TSS around the Dirac point as well^28^. As Sb_2_Te_2_Se and Bi_2_Se_3_ comprise different elements, their wave functions must have different orbital characters. Such a difference further implies that the *g*-factors of the two compounds should be different too. Note that a considerable energy dependence of the *g*-factor is expected, since the orbital character of the wave function changes notably in these systems. Our measurement is merely around the Dirac point, which is directly relevant to the gap opening of the TSS via *B*. In this regard, the *g*-factor of the TSS may be different from that of the bulk since they are measured at different energies. Further theoretical investigations regarding those factors are needed to develop a general theory that describes the *g*-factor.

Considering the significant material dependence of *g*_s_, we envision an interesting possibility to tailor the *g*-factor of the TSS by controlling the chemical composition of the chalcogenide TI materials in the form of solid solutions^29^,^30^. This provides a new knob in manipulating the TSS for its spin-related applications.

## Methods

### Experiment description

The experiments were performed with a modified commercial UNISOKU low temperature STM at 4.4 or 1.5 K. Magnetic fields up to 12 T can be applied perpendicularly to the sample surface. Sb_2_Te_2_Se and Bi_2_Se_3_ crystals grown by a modified Bridgman technique were cleaved *in situ* under ultrahigh vacuum conditions at∼77 K. After cleaving, the crystals were transferred quickly to the low-temperature STM for subsequent measurements. Two Bi_2_Se_3_ and three Sb_2_Te_2_Se samples were measured. A tungsten tip was used as the STM probe, which had been cleaned and characterized with a field-ion microscope. The tunnelling spectra were obtained by lock-in detection of the tunnelling current with a modulation voltage at 617.3 Hz feeding into the sample bias. The tip was grounded to provide the reference voltage.

### Evaluation of the finite curvature of LL scaling

We evaluated m*, which characterizes the finite curvature, using the scaling analysis of the LL energies shown in Fig. 2c. Since only the Zeeman shift of the LL_0_ is prominent, Supplementary equation (10) (Supplementary Note 1) can be approximated by 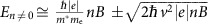. This indicates that the scaling of *E*_*n*_ with (*nB*)^1/2^ still applies even in the case of finite m*. Regarding *E*_0_, its energy at 3 T was used for the scaling analysis, because its shift is negligible at low *B*. By fitting the low-energy part of the scaling relation, the m* value can be obtained. It must be noted that the low-energy fitting considerably deteriorated when high-energy data were also included. Since the effect of m* on *E*_0_ is determined by the electronic states around the Dirac point, we only fitted the low-energy parts.

### Fitting the potential extremes with 2D parabolic potential model

First, two sectional lines across the potential extreme were drawn and fitted with a one-dimensional parabolic potential to estimate its shape and location. The obtained parameters were subsequently input as the initial guess of the 2D parabolic fitting. The 2D fitting provided the shape and location of the fitted potential, whose equipotential lines were superimposed on the potential map (Supplementary Figs 3a,b and 4a,b). The generated fitting error was small, demonstrating that the 2D parabolic potential model fitted the measured potential well. This conclusion was further augmented by evaluating two sectional lines extracted along the major eclipse axes of both the fitted potential and the measured *E*_0_ map (Supplementary Figs 3c–f and 4c–f). Since the 2D parabolic potential merely applies to potential extremes, it cannot be used at low *B*, where the LL_0_ state spatially expands beyond the potential extremes. (The effect of the potential variations at low *B* is discussed in Supplementary Note 3, Supplementary Figs 6 and 7). Therefore, for the fitting of Figs 3c,f and 4c,f, we did not include the data below the critical *B* value, where the potential-corrected *E*_0_ begins to deviate from the *B*-linear behaviour. The size of the LL_0_ state (2*l*_*B*_) at the critical *B* (Supplementary Figs 3a,b and 4a,b, dashed circles) has a good correspondence with the fitted area, which substantiates our model.

## Additional information

**How to cite this article:** Fu, Y.-S. *et al*. Observation of Zeeman effect in topological surface state with distinct material dependence. *Nat. Commun.* 7:10829 doi: 10.1038/ncomms10829 (2016).

## Supplementary Material

Supplementary Information Supplementary Figures 1-7, Supplementary Notes 1-3 and Supplementary References
