# Supplementary material for: Observation of Zeeman effect in topological surface state with distinct material dependence
Source: Nat Commun. 2016 Feb 24;7:10829. doi: 10.1038/ncomms10829 (PMC4770087; doi:10.1038/ncomms10829)
Supplement: Supplementary Information — Supplementary Figures 1-7, Supplementary Notes 1-3 and Supplementary References [file ncomms10829-s1.pdf]

SUPPLEMENTARY FIGURES

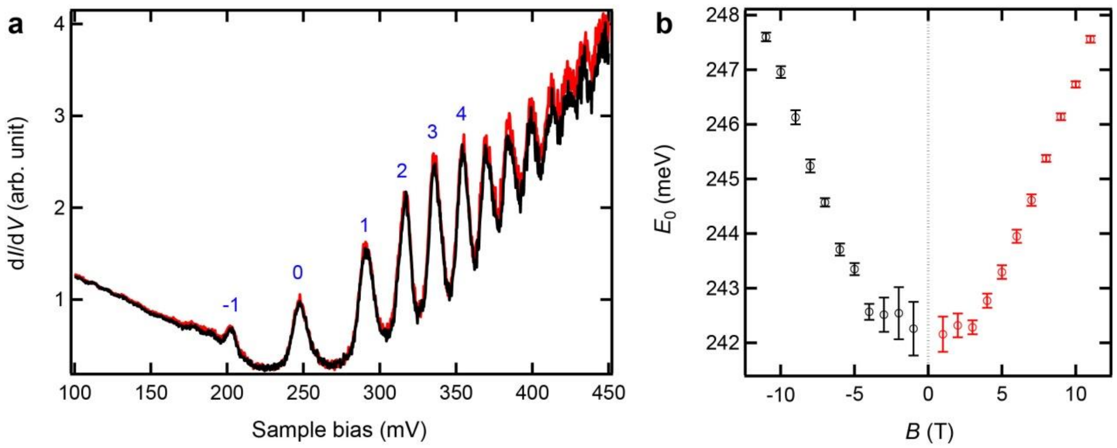

Supplementary Figure 1 LL spectra at positive and negative *B*. (**a**) Tunneling spectra measured at the same location of Sb_2Te_2Se showing identical LLs at positive (red line) and negative (black line) *B* of 11 T. The LL indexes are marked with blue numbers. Measurement conditions are V_s = -100 mV, I_t = 50 pA and V_{mod} = 1.4 mV_{rms}. (**b**) E_0 values of Sb_2Te_2Se extracted by fitting the LL_0 peak of **a** at various positive (red symbols) and negative (black symbols) *B* with a Lorentz shape. The error bars denote the standard errors estimated by the fitting.

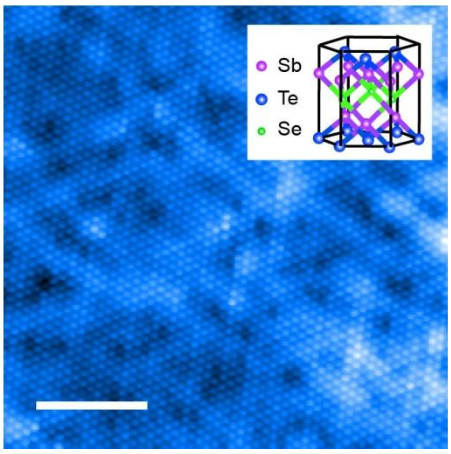

Supplementary Figure 2 Atomic resolution STM image showing the topography of Sb_2Te_2Se surface. Imaging condition: V_s = 250 mV and I_t = 10 pA. The insert depicts the crystal structure of Sb_2Te_2Se. The scale bar corresponds to 5 nm.

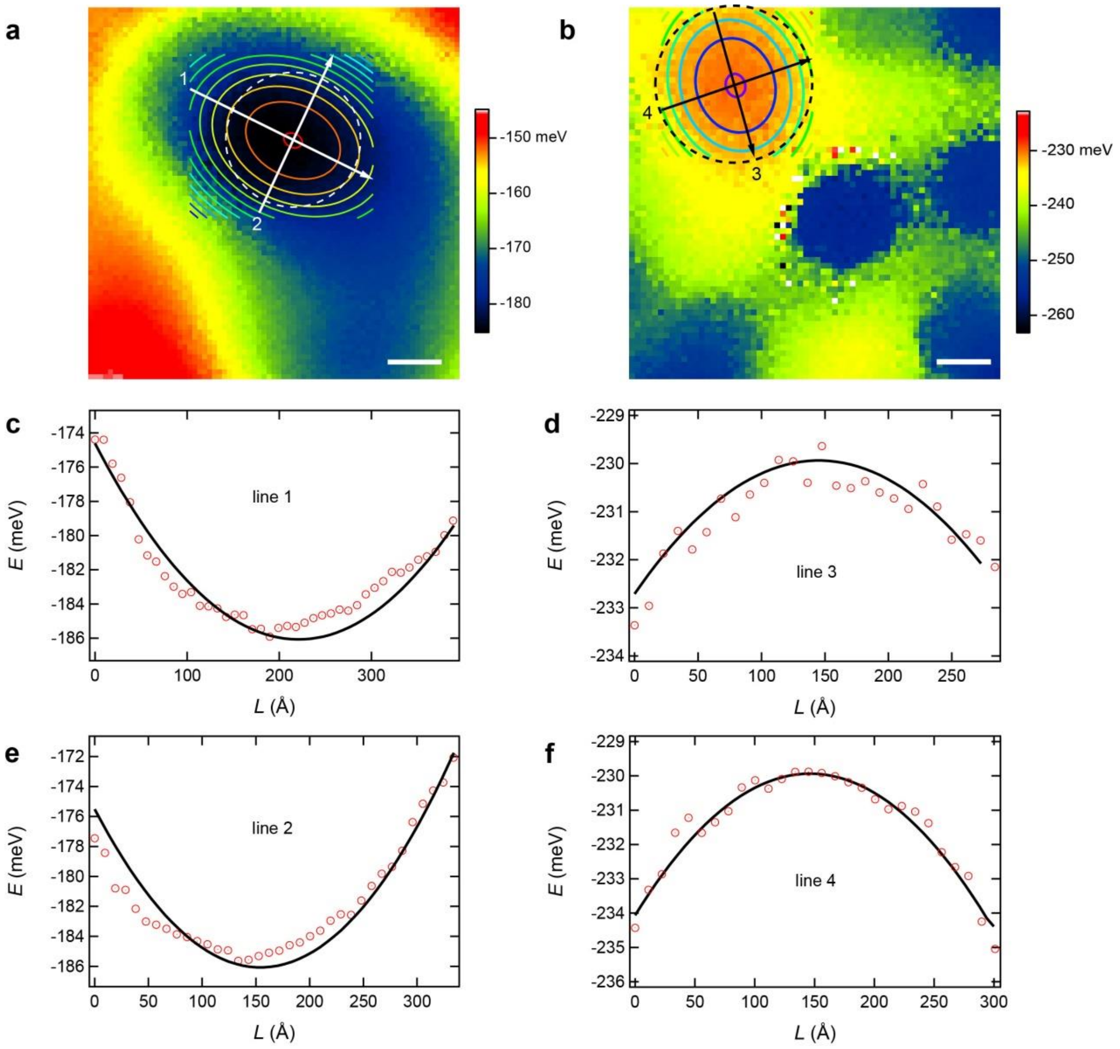

Supplementary Figure 3 2D parabolic potential fitting to Bi_2Se_3. (**a**,**b**) Potential maps of Bi_2Se_3 surface obtained by the spectroscopic imaging of E_0 at 11 T. The potential maps are the same as those of Fig. 3 in main text. The potential extremes are fitted with the 2D parabolic potential model. The solid ellipses represent the equipotential lines of the fitted potential. The adjacent potential lines have an energy interval of 2 meV for **a** and 1meV for **b**. The innermost ellipse corresponds to -186 meV (-230 meV) for **a** (**b**). The scale bar corresponds to 10 nm. (**c**-**f**) Sectional lines extracted from the E_0 map (red symbols) and the fitted potential (black curves) along the major (lines 1 and 3) and minor axes (lines 2 and 4) of the fitted equipotential ellipses. The data in **c** and **e** are extracted from **a**. The data in **d** and **f** are extracted from **b**. The dashed circle in **a** (**b**) characterizes the location and size of the LL_0 state at 4 T (3 T).

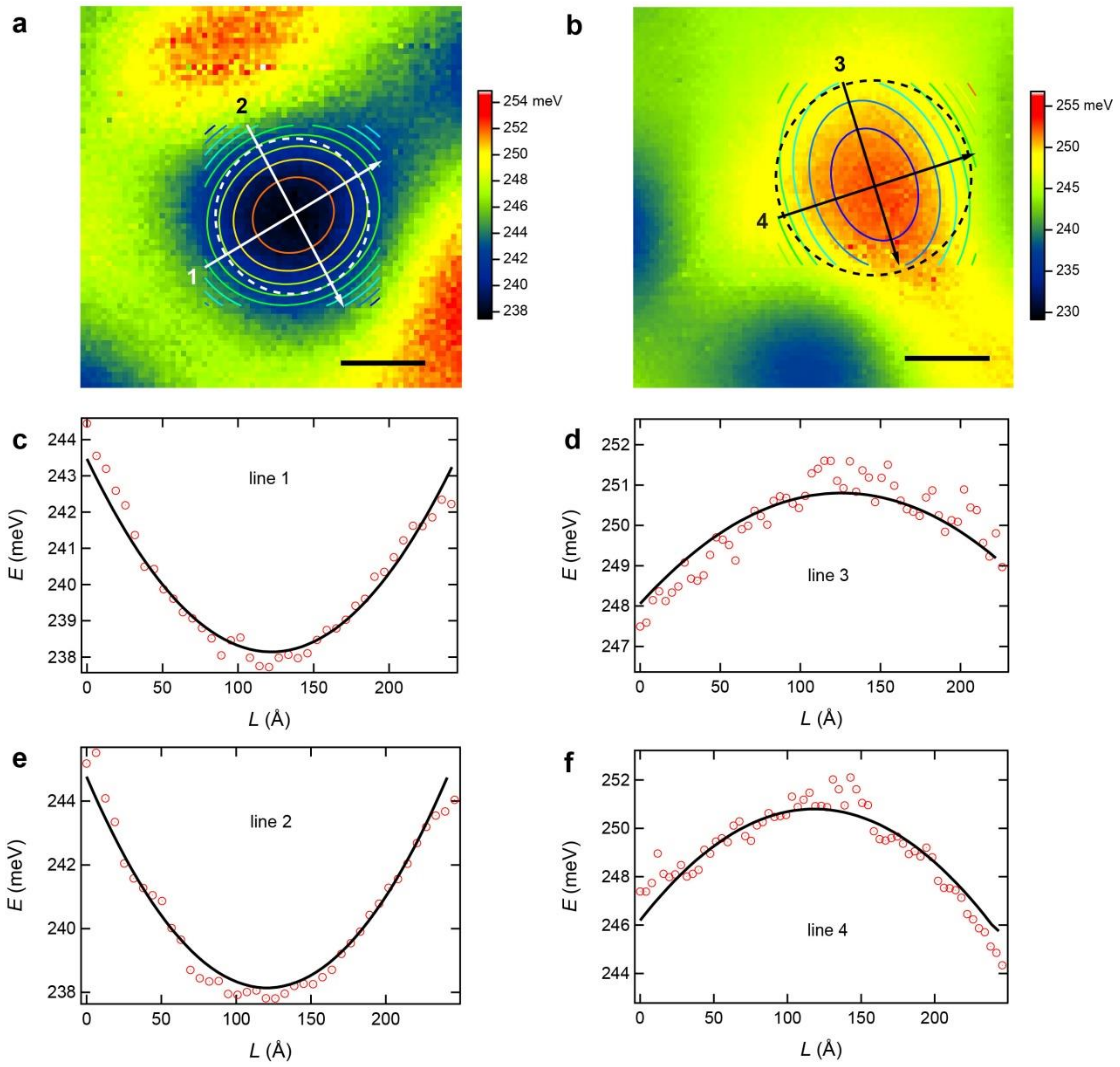

Supplementary Figure 4 2D parabolic potential fitting to Sb_2Te_2Se. (**a,b**) Potential maps of Sb_2Te_2Se surface obtained by the spectroscopic imaging of E_0 at 12 T. The potential maps are the same as those of Fig. 4 in main text. The potential extremes are fitted with the 2D parabolic potential model. The solid ellipses represent the equipotential lines of the fitted potential. The adjacent potential lines have an energy interval of 1meV. The innermost ellipse corresponds to 239 meV (250 meV) for **a** (**b**). The scale bar corresponds to 10 nm. (**c-f**) Sectional lines extracted from the E_0 map (red symbols) and the fitted potential (black curves) along the major (lines 1 and 3) and minor axes (lines 2 and 4) of the fitted equipotential ellipses. The data in **c** and **e** are extracted from **a**. The data in **d** and **f** are extracted from **b**. The dashed circle in **a** (**b**) characterizes the location and size of the LL_0 state at 8 T (5 T).

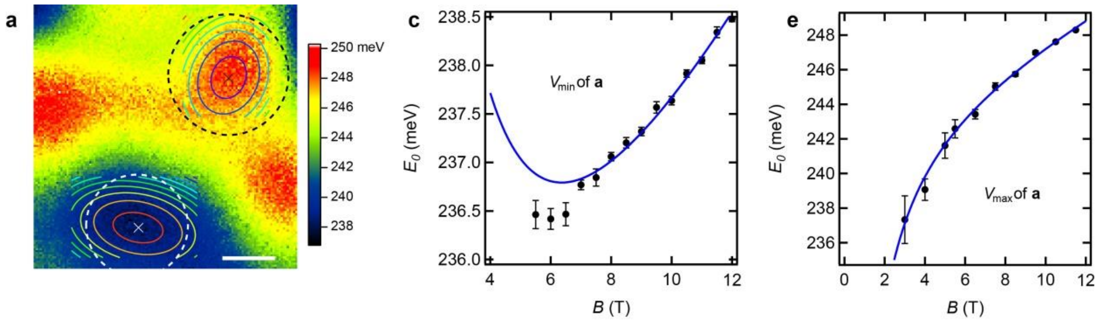

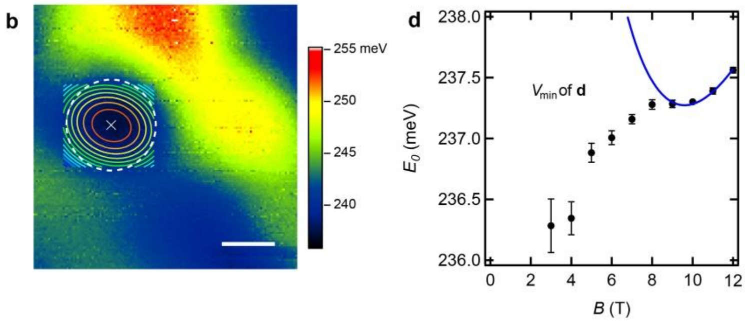

f

potential	α_x (meV/nm²)	α_y (meV/nm²)	$\frac{2}{m^*} - g_s$
V_{min} Fig.4a	3.57	4.55	22.84
V_{max} Fig.4d	-3.30	-1.74	22.85
V_{min} of **a**	3.33	5.38	21.48
V_{max} of **a**	-2.36	-1.47	20.78
V_{min} of **b**	1.02	3.06	22.89

Supplementary Figure 5 Measuring the *g*-factor of Sb_2Te_2Se at several potential extremes. Potential maps of the Sb_2Te_2Se surface obtained by the spectroscopic imaging of E_0 at 12 T showing a potential minimum (V_{min}) and maximum (V_{max}) in **a** and a single potential minimum in **b**. The scale bar corresponds to 10 nm. The solid ellipses represent the equipotential lines of the fitted 2D parabolic potential. The innermost ellipse corresponds to 248 meV for V_{min} in **a**, 238.5 meV for V_{max} in **a** and 238meV in **b**. The adjacent equipotential lines have an energy interval of 0.5 meV. The dashed circles in **a** and **b** characterize the location and size of the LL_0 state at 7 T for V_{min} of **a**, 5 T for V_{max} of **a**, and 9 T for V_{min} of **b**. (**c**, **d**, **e**) E_0 at different B (black symbols) measured at the fitted potential extremes (marked as crosses in **a** and **b**) and their fitting according to Eq. 2 of main text (blue curves). The error bars of E_0 are the standard of deviation generated from the LL_0 peak fitting with a Lorentz line shape. (**f**) Table showing the fitting results of the shape of the potential extremes and the values of $\frac{2}{m^*} - g_s$ including those shown in Fig. 4 of main text.

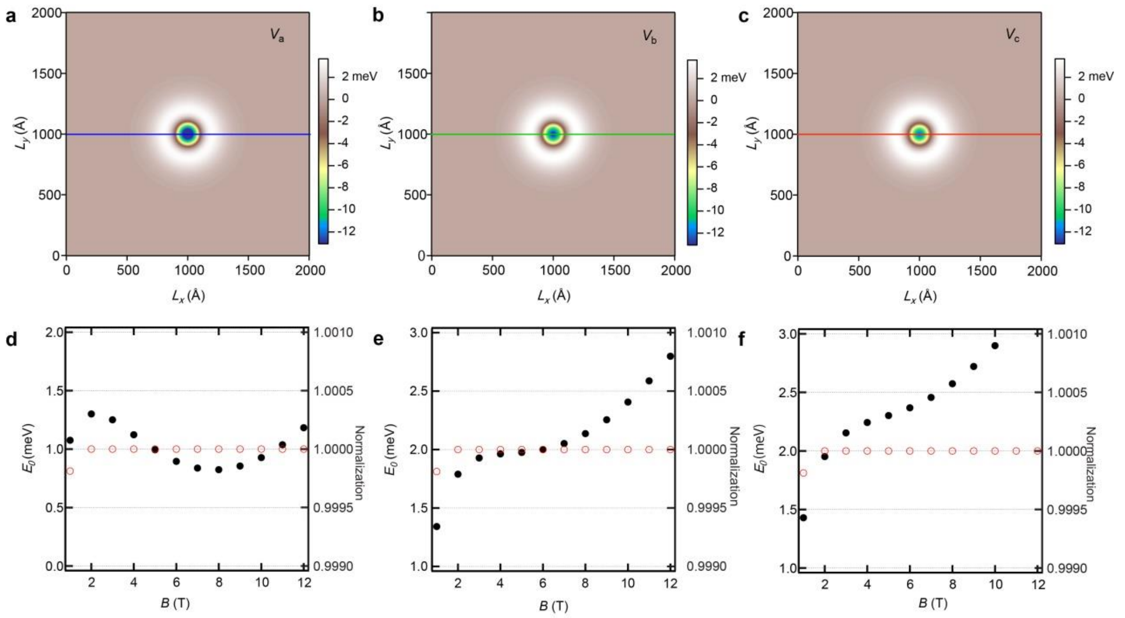

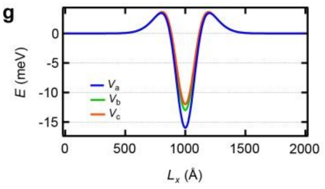

h

potential		V_a (meV)		V_b (meV)		V_c (meV)	
A_1 (meV)	A_2 (meV)	0.5	1.3	0.5	1.15	0.5	1.1
$(x_0, y_0)_1$ (Å)	$(x_0, y_0)_2$ (Å)	(1000, 1000)	(1000, 1000)	(1000, 1000)	(1000, 1000)	(1000, 1000)	(1000, 1000)
$(\sigma_x, \sigma_y)_1$ (Å)	$(\sigma_x, \sigma_y)_2$ (Å)	(160, 160)	(80, 80)	(160, 160)	(80, 80)	(160, 160)	(80, 80)

Supplementary Figure 6 Model calculations based on a single potential. (**a-c**) Single potentials with different shapes that are all a superposition of a Gaussian maximum and a Gaussian minimum. Pixel size: 512×512. (**d-f**) Calculated E_0 (black symbols) and the normalization value (red symbols) of the LL_0 state at different B according to Supplementary Eq. 12 and 13. (**g**) Sectional lines of Potential a-c across the potential center (horizontal lines in **a-c**). (**h**) Table showing parameters of Potential a-c. The different parameters among the three potentials are highlighted with the blue color.

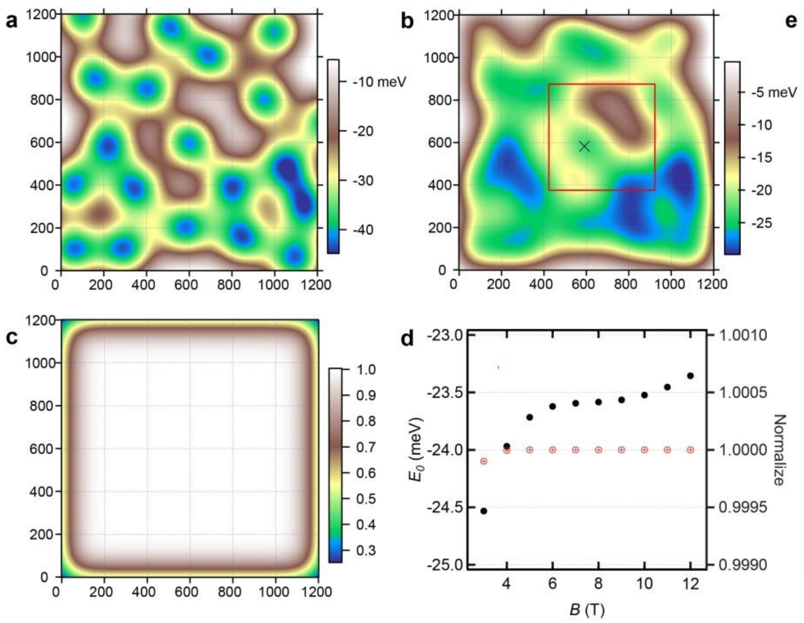

e

i	(x_i, y_i)	i	(x_i, y_i)
1	(600,600)	11	(700,1000)
2	(150,900)	12	(950,800)
3	(410,850)	13	(300,100)
4	(500,1150)	14	(1250,700)
5	(100,1200)	15	(1100,50)
6	(220,600)	16	(580,200)
7	(350,380)	17	(1000,1120)
8	(800,400)	18	(850,150)
9	(1050,500)	19	(100,400)
10	(1150,300)	20	(50,100)

Supplementary Figure 7 Model calculations based on multi-potential minimums. (**a**) A multi-minimum potential composed of 20 identical Gaussian minimums. (**b**) Calculated E_0 map at 12 T according to Supplementary Eq. 14. The red rectangle corresponds to the modeled potential map of Supplementary Fig. 5b. (**c**) Calculated normalization map of the LL_0 state at 12 T. (**d**) Calculated E_0 (black symbols) and the normalization values (red symbols) of the LL_0 state at the potential minimum center of the rectangle area (marked as a cross in **b**) at different B. (**e**) Table showing the parameters of the Gaussian potential minimums for constructing the potential in **a**. Pixel size of **a-c**: 512×512. The value for $\frac{2}{m^*} - g_s$ is 20 during the calculation.

SUPPLEMENTARY NOTES

Supplementary Note 1 - Models of LLs in TSS in the presence of Zeeman effect

We consider two models for the TSS and study their LLs in the presence of the Zeeman effect: one is the ideal helical Dirac fermions, another one is the non-ideal helical Dirac fermions perturbed by a parabolic curvature in their energy dispersion and the potential variation.

Ideal helical Dirac fermions

The Hamiltonian for the ideal helical Dirac fermions in a perpendicular magnetic field B is given as:

$$H = v(\sigma_x \Pi_y - \sigma_y \Pi_x) + \frac{1}{2} g_{\mathrm{s}} \mu_{\mathrm{B}} B \sigma_z \tag{1}$$

Here, $\mathbf{\Pi} = \hbar \mathbf{k} + |e| \mathbf{A}$ is the canonical momentum, with $\mathbf{k}$ and $\mathbf{A}$ being the momentum and the vector potential, respectively; v is the electron velocity; $\boldsymbol{\sigma}$ are the Pauli matrices, and g_{s} is the electron g-factor of the TSS. We assume $B = |\mathbf{B}|$ throughout the paper. After introducing the ladder operators $a = \frac{l_B}{\sqrt{2}\hbar}(\Pi_y + i\Pi_x)$ and $a^\dagger = \frac{l_B}{\sqrt{2}\hbar}(\Pi_y - i\Pi_x)$, the Hamiltonian is reformulated as

$$H = \frac{\sqrt{2}\hbar v}{l_B} \begin{pmatrix} 0 & a \\ a^\dagger & 0 \end{pmatrix} + \frac{1}{2} g_{\mathrm{s}} \mu_{\mathrm{B}} B \begin{pmatrix} 1 & 0 \\ 0 & -1 \end{pmatrix} \tag{2}$$

Since the wave function is a 2-spinor $\psi_n = \begin{pmatrix} u_n \\ v_n \end{pmatrix}$, then we get

$$\frac{1}{2} g_{\mathrm{s}} \mu_{\mathrm{B}} B u_n + \frac{\sqrt{2}\hbar v}{l_B} a v_n = E_n u_n \tag{3}$$

$$\frac{\sqrt{2}\hbar v}{l_B} a^{\dagger} u_n - \frac{1}{2} g_{\mathrm{s}} \mu_{\mathrm{B}} B v_n = E_n v_n \qquad (4)$$

For ladder operators, $a^{\dagger}a|n\rangle = n|n\rangle$, $a^{\dagger}|n\rangle = \sqrt{n+1}|n+1\rangle$, $a|n\rangle = \sqrt{n}|n-1\rangle$, where n is a non-negative integer. This, in combination with Supplementary Eq. 3 and 4, yields

$$E_{n\neq 0} = \pm\sqrt{2|e|\hbar v^2 nB + (g_{\mathrm{s}}\mu_{\mathrm{B}}B/2)^2} \qquad (5)$$

$$\begin{cases} \psi^{+}{}_{n\neq 0} = \sqrt{\dfrac{1}{1+D_n{}^2}} \begin{pmatrix} D_n|n-1\rangle \\ |n\rangle \end{pmatrix} \\ \psi^{-}{}_{n\neq 0} = \sqrt{\dfrac{1}{1+D_n{}^2}} \begin{pmatrix} |n-1\rangle \\ -D_n|n\rangle \end{pmatrix} \end{cases} \qquad (6)$$

where $D_n = \dfrac{1}{\sqrt{1+\dfrac{(g_{\mathrm{s}}\mu_{\mathrm{B}}B/2)^2}{2|e|\hbar v^2 nB}} - \dfrac{g_{\mathrm{s}}\mu_{\mathrm{B}}B/2}{\sqrt{2|e|\hbar v^2 nB}}}$. The positive (negative) brunch represents the electrons (holes) of the Dirac fermions.

Because $a|0\rangle = 0$, we get

$$E_{n=0} = -g_{\mathrm{s}}\mu_{\mathrm{B}}B/2 \text{, and } \psi_{n=0} = \begin{pmatrix} 0 \\ |0\rangle \end{pmatrix} \qquad (7)$$

It is seen from Supplementary Eq. 5 and 7 that the Zeeman shift of LL_n can be estimated as $\Delta E_{n\neq 0} \cong \pm\dfrac{(g_{\mathrm{s}}\mu_{\mathrm{B}}B/2)^2}{\sqrt{2|e|\hbar v^2 nB}}$. Evidently, the Zeeman shift is largest for the LL_0 state and decreases dramatically for LL_n with increasing n. The energy-resolved spin magnetization is defined as $m_i = \frac{\hbar}{2}\langle\psi_n|\sigma_i|\psi_n\rangle$, $i = x, y, z$. Hence, the spin magnetization of the ideal helical Dirac fermions can be calculated using the LL wave functions given in Supplementary Eq. 6 and 7. While the in-plane spin

magnetization is determined to be zero, the out-of-plane spin magnetization is $m_{z,n=0} = -\frac{\hbar}{2}$ and $m_{z,n\neq 0} = \pm\frac{\hbar}{2}\cdot\frac{D_n^2-1}{D_n^2+1}$. Thereby, m_z decreases rapidly with increasing n as well.

We further evaluate the situation of reversing the direction of *B*. Applying a negative *B* to the TI is equivalent to probing its opposite surface in a positive *B* [1]. For the negative *B*, the Hamiltonian becomes:

$$H = v(\sigma_x\Pi_y - \sigma_y\Pi_x) - \frac{1}{2}g_s\mu_B B\sigma_z \qquad (8)$$

The ladder operators should accordingly change to $a = \frac{l_B}{\sqrt{2}\hbar}(\Pi_y - i\Pi_x)$ and $a^\dagger = \frac{l_B}{\sqrt{2}\hbar}(\Pi_y + i\Pi_x)$. Following similar algebra as the case of the positive *B*, we obtain the same expressions for the LL energies as Supplementary Eq. 5 and 7. This is different from the conclusion of Supplementary Ref. 2, which claims that the Zeeman shift of E_0 is dependent on the applied direction of the perpendicular *B*. Our expectations are experimentally justified by measuring the LLs of the TSS in both positive and negative *B* (Supplementary Fig. 1). Regarding the LL wave functions, their upper and lower components switches when the sign of *B* changes. Consequently, m_z of the helical Dirac fermions also reverses their sign.

Non-ideal helical Dirac fermions

Actual TSSs are not ideal because their energy dispersions have a finite curvature and there exist potential variations spatially as well. Those two factors should be properly considered in the Hamiltonian. Firstly, to describe the finite band curvature, a parabolic term is introduced to the Hamiltonian of Supplementary Eq. 1, which then becomes:

$$H = \frac{1}{2m^* m_e}(\Pi_x{}^2 + \Pi_y{}^2) + v(\sigma_x \Pi_y - \sigma_y \Pi_x) + \frac{1}{2} g_s \mu_B B \sigma_z \qquad (9)$$

Where m_e and m^* are the absolute and relative effective mass of the electrons, respectively. The expressions of its LL energies have been given in Supplementary Ref. 3 as:

$$E_{n \neq 0} = \hbar\omega_c n \pm \sqrt{2\hbar v^2 |e| nB + (\frac{1}{2}\hbar\omega_c - \frac{1}{2} g_s \mu_B B)^2}$$

$$E_{n=0} = \frac{1}{2}\hbar\omega_c - \frac{1}{2} g_s \mu_B B = \frac{1}{2}(\frac{2}{m^*} - g_s)\mu \qquad (10)$$

Where $\omega_c = \frac{|e| B}{m^* m_e}$ is the cyclotron frequency of the parabolic electrons.

Next, we model the influence of the potential variations on the LL energies. To approximate the potential shape, we use a 2D parabolic potential model, which is $V(x, y) = E_D + \alpha_x x^2 + \alpha_y y^2$. At the potential extreme, the energy shift of E_0 caused by the potential variations is given analytically to a first approximation as [4]:

$$E_V = \iint \phi_0 V \phi_0 dxdy = E_D + (\alpha_x + \alpha_y) l_B{}^2 = E_D + (\alpha_x + \alpha_y)\frac{\hbar}{|e| B} \qquad (11)$$

Where $\phi_0 = \frac{1}{\sqrt{2\pi} l_B} \exp(-\frac{x^2 + y^2}{4 l_B{}^2})$ is the wave function of the LL_0 state. Consequently, the E_0 value considering the Zeeman shift, the finite m^* and the potential variation can be obtained by combining Supplementary Eq. 10 and 11, and is conclusively written as Eq. 2 of main text.

Supplementary Note 2 - Topography of Sb_2Te_2Se surface

Sb_2Te_2Se has the tetradymite structure, which is identical to that of Bi_2Se_3. Its quintuple-layer unit consists of Te-Sb-Se-Sb-Te (Supplementary Fig. 2, insert). The bonding forces between the quintuple layers are weak van der Waals interactions. Therefore, the crystal cleaves easily. STM image of the cleaved surface clearly resolves the ordered atoms of the triangular lattice (Supplementary Fig. 2). Its lattice constant is estimated to be 4.2 Å, which is consistent with the bulk value. Since cleaving occurs between the adjacent Te layers, the imaged atoms should be Te.

Supplementary Note 3 - Modeling the effect of potential extensions on E_0 at low B

In the TSS of Sb_2Te_2Se, the non-ideal dispersions and the Zeeman effect both make E_0 shift towards higher energy with B. In contrast, the effect of a potential minimum make E_0 shift oppositely with B. As a result, E_0 first decreases and then increases with B at a potential minimum, as is seen from Fig. 4c. However, the shifting trend of the $E_0(B)$ differs at different potential minimums. For instance, E_0 exhibits a monotonic B-shift at the potential minimum of Supplementary Fig. 5d. As the spatial extension of the LL_0 state expands at low B, the effect of the potential at large extensions takes place. In this section, we model this effect to understand the observed diverse shifting behavior of E_0 at different potential minimums.

We first simply use two superimposed Gaussian potentials to model the potential minimum, because they contain different types of variations and become flat at large extensions. The Gaussian potential writes $V^{G}(x,y) = A\exp(-(\frac{(x-x_0)^2}{2\sigma_x^{\,2}}+\frac{(y-y_0)^2}{2\sigma_y^{\,2}}))$, where A is the amplitude, (x_0, y_0) is the center and (σ_x, σ_y) is the decay length of the potential. The model potential is $V(x,y) = V^{G}{}_1 - V^{G}{}_2$. We modeled three potentials (Supplementary Fig. 6a-c) to elucidate the different shifting behavior of $E_0(B)$. Their parameters are listed in Supplementary Fig. 6h. The three potentials all have a dip in

the center, and decrease in energy after reaching a maximum as their sizes spatially extend, thereby forming a hump shape. The depth of the potential dip is deepest for Potential a (V_a), and shallowest for Potential c (V_c) (Supplementary Fig. 6g).

Subsequently, the E_0 value can be calculated according to Supplementary Eq. 10 and 11 as

$$E_0 = \iint \phi_0 V \phi_0 dxdy + \frac{1}{2}(\frac{2}{m^*} - g_s)\mu_B B \qquad (12)$$

Where $\phi_0 = \frac{1}{\sqrt{2\pi} l_B} \exp(-\frac{x^2 + y^2}{4{l_B}^2})$ is the wave function of LL_0 state. The first term depicts the potential effect, and the second term represents the influence from the non-ideal dispersion and the Zeeman effect. We use a value of 20 for $\frac{2}{m^*} - g_s$, which is close to the experimentally measured value of 19. The integration is calculated in the range of $[x, y] = [(0, 2000), (0, 2000)]$. To guarantee the LL_0 state at all calculated B is within the integration range, we always check its normalization value is one or not (Supplementary Fig. 6d-f), which is given as

$$N_0 = \int_0^{2000} \int_0^{2000} |\phi_0|^2 \, dxdy \qquad (13)$$

The calculated $E_0(B)$ exhibit different shifting trends at different potential minimum centers (Supplementary Fig. 6d-f). Such differences can be interpreted from the weighting of the LL wave functions at the potentials. When the potential dip is deep (Potential a), the LL_0 state is mostly weighted by the potential dip even at low B. Therefore, its E_0 first decreases and then increases with decreasing B (Supplementary Fig. 6d), as is expected for a potential minimum shown in Fig. 4c. It must be noted that the normalization value of the LL_0 state at 1 T is less than 1, which means its calculated E_0 should be neglected. When the potential dip is shallow (Potential b), the LL_0 state significantly enhances its weighting at the potential hump. This makes E_0 shift monotonically with decreasing B (Supplementary Fig. 6e) in a similar manner as

Supplementary Fig. 5d. The monotonic B-shifting behavior of E_0 is more evident (Supplementary Fig. 6f) as the potential dip gets even shallower (Potential c).

On the basis of the single potential indicated above, we further construct a multi-minimum potential to reproduce the actual potential variations shown in Supplementary Fig. 5b. Supplementary Fig. 7a shows the modeled potential, which is composed of 20 Gaussian potentials with identical shapes but different coordinate centers, i.e.

$$V(x,y)=\sum_{i=1}^{20} V^{\mathrm{G}}{}_{i}(x,y) \qquad (14)$$

Where $V^{\mathrm{G}}{}_{i}(x,y)=2\exp(-(\frac{(x-x_i)^2}{2\times100^2}+\frac{(y-y_i)^2}{2\times100^2}))$ is the single Gaussian potential, and the parameters of (x_i, y_i) are listed in Supplementary Fig. 7e.

Then, the E_0 map at 12 T can be obtained by calculating E_0 at every pixel point according to Supplementary Eq. 12 (Supplementary Fig. 7b). Since our integration range is limited to $[x,y]=[(0,1200),(0,1200)]$, we further calculate the normalization values of the LL_0 state at every pixel point according to Supplementary Eq. 13 (Supplementary Fig. 7c). The E_0 values of the boundary regions, whose normalization values are smaller than 1, should be neglected. The calculated E_0 map (Supplementary Fig. 7b, red rectangle) reproduces the measurement of Supplementary Fig. 5b well. We then calculate E_0 of the potential minimum (Supplementary Fig. 7b, cross) at different B (Supplementary Fig. 7d). The obtained shifting trend reproduces that of Supplementary Fig. 5d as well. Therefore, our model calculations substantiate our experimental observations, demonstrating that the potential at large extensions could not only affects the amount of shifting of E_0 with B but also changes its trend.

Supplementary References:

1. Shen, S.Q. Quantum Hall effect of the surface states in topological insulator. Preprint at http://arxiv.org/abs/0909.4125 (2009).

2. Vazifeh, M. M. & Franz, M. Spin response of electrons on the surface of a topological insulator. *Phys. Rev. B* **86**, 045451 (2012).

3. Reynoso, A., Usaj, G., Sánchez, M. J. & Balseiro, C. A. Theory of edge states in systems with Rashba spin-orbit coupling. *Phys. Rev. B* **70**, 235344 (2004).

4. Okada, Y. *et al*. Visualizing Landau levels of Dirac electrons in a one-dimensional potential. *Phys. Rev. Lett.* **109**, 166407 (2012).
